# A Tale of Two Brothers: Familial Voltage-Gated Potassium Channel Autoimmune Encephalitis

**DOI:** 10.7759/cureus.8723

**Published:** 2020-06-20

**Authors:** Lauren E Gillespie, Amanda Dave, Amy Goldstein

**Affiliations:** 1 Pediatrics, Creighton University School of Medicine, Omaha, USA; 2 Pediatrics, University of Nebraska Medical Center, Omaha, USA

**Keywords:** vgkc encephalitis, autoimmune encephalitis, lgi1, caspr2, channelopathies, vgkc, encephalitis, voltage-gated potassium channel autoimmune encephalitis, autoimmune, neuroimmunology

## Abstract

This is the first reported case of familial voltage-gated potassium channel (VGKC) autoimmune encephalitis. The symptoms of autoimmune encephalitis can mimic infectious encephalitis with headache, fatigue, and neuropsychiatric symptoms. Autoimmunity is emerging as a distinct cause of encephalitis in the children. Prompt recognition, diagnosis, and treatment are important to prevent brain damage. Two brothers presented two years apart with different symptoms. The explanation for their distinct symptoms lies in the multifactorial development of autoimmunity. The presentation of autoimmune encephalitis can depend on the offending antibodies. The most common are antibodies against the N-methyl-D-aspartic acid (NMDA) receptor and the VGKC complex. Antibodies to the VGKC complex are divided into three different groups depending on their antigenic target: leucine-rich glioma-inactivated protein 1 (LGI1), contactin-associated protein-like 2 (CASPR2), or neither. Anti-VGKC antibodies in children are associated with neuroinflammation and encephalitis. Autoimmunity to LGI1 and CASPR2 antigens is associated with distinct human leukocyte antigen (HLA) alleles. Different HLA isotypes are involved in antigen processing and presentation and can lead to a genetic predisposition to autoimmunity. VGKC autoimmune encephalitis can present with memory changes, psychiatric symptoms, and motor abnormalities. Both brothers presented with these symptoms in their own unique way. Efficient diagnosis and immunosuppression helped improve their outcomes.

## Introduction

This is the first documented case of familial voltage-gated potassium channel (VGKC) autoimmune encephalitis. VGKCs are important ion channels that regulate neuron action potentials. Dysfunction in the channel prolongs cell action potential, which can lead to seizures, cerebellar ataxia, encephalitis, neuropsychiatric symptoms, and other clinical manifestations [1]. VGKC autoimmune encephalitis has been shown to come from an antibody targeting the cell surface antigen. Infectious and autoimmune encephalitis can present in a similar manner, but serum and cerebrospinal fluid (CSF) analysis can help determine the etiology. For autoimmune channelopathies, the BrainWorks treatment protocol has guidelines for proper immunosuppression and monitoring. The two patients in this case series presented with distinct symptoms within various timelines and responded differently to the treatment protocol.

Informed consent was obtained by the patient’s legal guardians to write this case.

## Case presentation

Case no. 1

**Figure 1:**
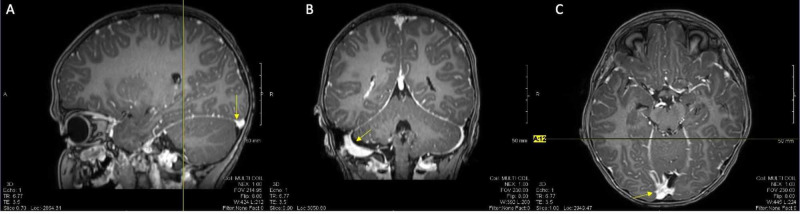
Initial brain MRI of patient 1 Evidence of hyperenhancement, portions identified with yellow arrows in the respective planes. (A) Sagittal view, (B) coronal view, (C) axial view.

Patient 1 is a seven-year-old male who presented in the fall of 2016 with a four-day history of nausea, vomiting, headache, nuchal rigidity, and altered mental status. Relevant history includes attention deficit hyperactivity disorder (ADHD), developmental speech, and motor delays. Physical exam was significant for dilated pupils, flaccid hemiplegia affecting left nondominant side, dysphagia, inability to speak, agitation, and abnormal involuntary movements. There were no focal findings on exam to suggest a stroke, bleed, or mass. Infectious disease and neurology were consulted. Nasogastric feeds were initiated to support his nutrition due to his dysphagia. Brain MRI showed hyperenhancement of the meninges, cortical vessels, and subarachnoid spaces as noted in Figure 1. MRI and electroencephalogram (EEG) did not indicate a specific form of encephalopathy. His infectious studies were negative, and his autoimmune encephalopathy panel was positive for anti-VGKC antibodies.

Table 1 summarizes pertinent tests results. Patient 1 was admitted for 14 days. The patient received high-dose Solu-Medrol and intravenous immunoglobulins (IVIG) per the BrainWorks protocol with subsequent improvement of encephalopathy. He was discharged on a steroid taper. He also developed benzodiazepine and opiate dependence during his hospitalization, and a taper was instituted to avoid withdrawal. His nutrition was supported with nasogastric tube feedings. At time of discharge, the patient was able to speak coherently but his language was delayed for age. Additionally, his strength was improving and symmetric. The patient was discharged to inpatient rehabilitation to facilitate physical and occupational therapy. On recent follow-up, his serum titers are now negative.

Case no. 2

Patient 2 (patient 1’s biological sibling) presented two years later as an eight-year-old male with altered mental status described as confusion, as well as auditory and visual hallucinations. Five to six weeks earlier, he developed a sensation of bugs crawling on him, a symptom that did not seem to be a problem until two days before he presented to the emergency department. The patient exhibited intense fear of being alone, which contributed to an episode of enuresis. The patient’s medical history included ADHD managed with methylphenidate. He also received cyproheptadine for appetite stimulation due to poor weight gain.

Patient 2 was admitted and an encephalitis workup occurred. Serum and CSF samples were sent for evaluation and are described in Table 1. An EEG and MRI were performed and read as normal. Unfortunately, an insufficient CSF sample for the Mayo Clinic Encephalopathy-Autoimmune Evaluation, CSF was taken from the patient. However, the serum autoimmune encephalitis panel was positive. On day 2 of his hospital stay, his mental status and coordination declined precipitously. He continued to have episodes of hallucinations, paranoia, suicidal ideation, agitation, and irritability. For the encephalitis, he received IVIG and high-dose Solu-Medrol with a prednisone taper per the BrainWorks protocol. During his hospital stay, he received physical and occupational therapy. He was discharged on day 7 to inpatient rehabilitation to facilitate physical and occupational rehabilitation. After completion of rehabilitation, he appeared to be resistant to IVIG and rituximab was initiated. He continues to have agitation and episodes of psychosis and is still followed by neurology.

**Table 1:** Pertinent laboratory studies and imaging results for patient 1 and patient 2. * Lab values on initial evaluation. ** For patient 1, serum autoimmune panel not needed because Mayo Clinic Encephalopathy-Autoimmune Evaluation, CSF was positive for anti-VGKC. *** For patient 2, there was trouble getting enough CSF sample, so the patient was triaged. The CSF sample was insufficient for the cell count with differential test and the Mayo Clinic autoimmune encephalitis panel. CBC, complete blood count; CMP, comprehensive metabolic panel; CSF, cerebrospinal fluid; EEG, electroencephalogram; PCR, polymerase chain reaction; VGKC, voltage-gated potassium channel; WBC, white blood count; WNL, within normal limits.

	Test	Patient 1	Patient 2
Laboratory Studies	CMP*	WNL	WNL
	CBC*	WBC 12.04 x 10^3^/µL, neutrophils 79.7, lymphocytes 12.8	WNL
	Liver and kidney	Liver function test*: WNL	Urine drug screen*: positive for tricyclic antidepressants
	Mayo Clinic Encephalopathy, Autoimmune Evaluation, Serum	None**	Positive for anti-VGKC antibodies
Cerebrospinal Fluid Studies	Glucose	48 mg/dL	53 mg/dL
	Protein	52 mg/dL	19 mg/dL
	Culture	No growth	No growth
	Cell count with differential	Pleocytosis WBC 11 x 10^3^/µL	Insufficient sample***
	Virus	West Nile IgM: negative	Herpes simplex 1/2 PCR: negative. Arbovirus: negative
	Mayo Clinic Encephalopathy-Autoimmune Evaluation, CSF	Positive for anti-VGKC antibodies	Insufficient sample***
Brain Imaging	Initial MRI	Hyperenhancement of the meninges, cortical vessels, subarachnoid spaces	Normal
	Initial CT	None	No obvious mass nor hemorrhage noted, ventricles appear symmetric
	Follow-up MRI	Normal, 8 days later	None
	EEG	Normal	Normal

## Discussion

Childhood neuroimmune diseases of the central nervous system are overall rare conditions that have a wide variety of clinical presentations and result from dysregulation of the immune system. Categories of childhood neuroimmune disease include acquired demyelinating syndromes and autoimmune encephalitis [2]. Acquired demyelinating syndromes include acute disseminated encephalomyelitis, clinically isolated syndrome, neuromyelitis optica, and multiple sclerosis. Autoimmune encephalitis is caused by autoantibodies targeting antigens inside the neuron, on the cell surface, or in the extracellular synapse [3]. Encephalitis can result from an underlying antibody/complement-mediated immune response or T-cell-mediated cytotoxicity [3]. Autoimmunity can be triggered by a virus, be paraneoplastic, develop from a genetic predisposition, or arise sporadically [2,4]. Autoimmune encephalitis in children presents differently than adults, requiring a comprehensive clinical approach, development and adherence to child-specific encephalitis guidelines [4-7]. For neuroimmune diseases, targeted immunosuppression can stop or decrease the current immune insult on the body, reduce the likelihood of recurrence and long-term neuropsychiatric sequalae, and decrease morbidity and mortality. Clear communication with the patients and their parents on the illness trajectory and a multidisciplinary rehabilitation process can assist recovery and improve outcomes [8].

Encephalitis can present with seizures and neuropsychiatric symptoms associated with brain inflammation that develop over days to weeks [4,6]. If left untreated, encephalitis can progress to coma. It can be caused by infectious agents or be autoimmune. Etiologic discovery of encephalitis and other childhood neuroimmune diseases requires a detailed history of symptoms and presentation, a thorough physical exam, and laboratory studies. Helpful analysis includes serum studies, CSF studies, EEG of the brain, and MRI of the brain and spine [5,9]. Given the close presentation of infectious and autoimmune causes, empiric antiviral and antibacterial treatment is often started and then treatment is modified accordingly [9]. Prompt diagnosis and proper initiation of therapy can help improve long-term outcomes. 

A population based of children and adults found that the incidence and prevalence of autoimmune encephalitis are comparable to that of infectious encephalitis [10]. Infectious encephalitis is most often viral with causative agents, including herpes simplex virus 1 and 2, human herpes virus 6, Lyme disease, varicella zoster virus, enterovirus, Epstein Barr virus, West Nile virus, LaCrosse virus, *Ehrlichia*, toxoplasma, and John Cunningham virus [2,4,10]. Instances of *Bartonella henselae *encephalitis in pediatric populations have also been noted through case reports [11]. Infectious encephalitis had an annual incidence of 1.0/100,000 with a prevalence of 11.6/100,000. Autoimmune encephalitis had an annual incidence rate of 0.8/100,000 and prevalence of 13.7/100,000 [10]. The most common neuronal cell surface antigens include anti-N-methyl-D-aspartic acid (NMDA) receptors and anti-VGKC complex [3].

Autoimmunity is emerging as an important etiology of encephalitis in adults and children [3]. The detection of autoimmune encephalitis is increasing over time, in part due to an increase in neural-specific IgG-associated encephalitis, an increase in awareness in the clinical presentation, and the power of diagnostic tools such as next generation sequencing [10].

Treatment for autoimmune encephalitis includes high-dose corticosteroids with a taper, plasma exchange electrophoresis, and IVIG. Rituximab and cyclophosphamide are used in refractory cases [1,12]. This case used the BrainWorks protocol for antibody mediated channelopathies [12]. The BrainWorks protocol was established by the International Inflammatory Brain Disease Outcome Study through the Hospital for Sick Children (Toronto, ON, Canada) [13]. Both brothers received Solu-Medrol with a prednisone taper and IVIG. Patient 1 responded well to this regimen. He had negative serum titers and no neuropsychiatric or motor symptoms after treatment and rehabilitation. The younger brother, patient 2, continued to have neuropsychiatric symptoms after the first-line treatment and rehabilitation. He did not seem to be responsive to IVIG and then received rituximab for further immunosuppression.

Anti-NMDA receptor encephalitis is the most common type of antibody-mediated encephalitis overall and in children [1,14]. In children, it can cause headaches, fever, behavior problems, seizures, and movement disorders. It can be triggered after herpes simplex virus encephalitis resulting in choreoathetosis [14]. With immunosuppression, most children can have a full yet slow recovery [3].

The phenotype of VGKC encephalitis depends on which antigen is targeted. VGKC autoimmune encephalitis can be divided into three categories based on their main antigen within the protein complex: leucine-rich glioma-inactivated protein 1 (LGI1), contactin-associated protein-like 2 (CASPR2), or neither [15]. VGKC encephalitis with anti-LGI1 or CASPR2 antibodies is rare and is predominately found in male adults in their 60s [1]. Autoantibodies to LGI1 or CASPR2 can cause well-described clinical phenotypes in adults [1,15]. The hippocampus and limbic system is rich in expression in both LGI1 and CASPR2, so both subtypes of encephalitis can present with frequent focal seizures and amnesia. Patients with LGI-1 antibodies can have myoclonus, hyponatremia, and faciobrachial dystonic seizures. Those with CASPR2 antibodies can present as Morovan syndrome, which includes insomnia, neuropathic pain, peripheral nerve hyperexcitability, and neuropsychiatric features. There appears to be a slight genetic component related to human leukocyte antigen-DR isotype (HLA-DR). LGI1 autoantibodies are associated with HLA-DRB1*07:01, and CASPR2 autoantibodies are associated with HLA-DRB1*11:01 [15]. The identification of specific predisposing HLA isotypes likely implicate peptide binding and antigen presentation to B and T cells in disease initiation [15]. Given this genetic predisposition, further investigation is needed on how that influences the immunologic and environmental factors that trigger the autoimmune encephalitis process in adults and children.

In contrast to the distinct clinical presentations of anti-LGI1 or anti-CASPR2 encephalitis in adults, anti-VGKC antibodies in children form on varying epitopes of the membrane channel domain. Anti-VGKC antibodies in children do not indicate a specific clinical syndrome but instead are a nonspecific biomarker of neuroinflammation, particularly encephalopathy [16]. VGKC encephalitis in children is most often negative for both LGI1 and CASPR2 [16,17]. In this case, CSF and serum from both patients were sent out for the Mayo Clinic Encephalopathy-Autoimmune Evaluation testing. The Mayo Clinic Neuroimmunology Laboratory screened for the VGKC antibodies using the radioimmunoprecipitation assay [17]. In a recent 7.5-year span, serum samples from 13,319 pediatric patients was tested and 264 were positive for VGKC. Of those, only 13 were positive for either LGI1 or CASPR2 with a slight female predominance [17]. A few of the LGI1 and/or CASPR2-positive patients had preexisting autoimmunity, a diagnosis of functional neurological disorder, or neuropsychiatric symptoms. The dates of the Mayo Clinic Neuroimmunology Lab report did not overlap with this case, and therefore the data from this case are not included. It is unknown if the patients in this case study had antibodies to either LGI1 or CASPR2 antigens.

VGKC encephalitis can cause memory changes, psychiatric symptoms, and motor symptoms [5,7,18]. Imaging of those with VGKC autoimmune encephalitis can show a T2/fluid-attenuated inversion recovery (FLAIR) in the temporal lobe, consistent with limbic encephalitis [3]. Of note, the MRI of patient 1 did not reveal the cause of the encephalopathy and the MRI of patient 2 did not show any abnormalities. Both the brothers presented in this case have a history of ADHD, but their diagnosis was established before their respective acute encephalitis episodes. Patient 2 developed tactile hallucinations for five weeks before his symptoms worsened, and he presented to the hospital. He had more severe episodes of psychosis and he had higher VGKC antibody titers requiring additional treatment with rituximab. He continues to have episodes of psychosis likely due to his more severe initial presentation and resistance to first-line IVIG therapy.

In this case, the development and different clinical presentation of VGKC autoimmune encephalitis could have been caused by multiple factors. Because patient 1 and patent 2 are biological siblings, there is likely a genetic component to their development of VGKC autoimmune encephalitis. Their specific HLA subtypes and other genetic tests were not investigated at the time. Based on inheritance genetics as biological siblings, however, they have a 50% chance of sharing one HLA haplotype [19]. They could have a similar way of processing and presenting antigens, potentially leaving them with a genetic predisposition to develop this autoimmunity. Their antibodies could have targeted different antigens within the VGKC complex. Environmental factors likely played a role as well. The brothers were adopted by the same parents and they all live in one household environment. Their family stressors were different at the time when each brother developed the acute illness. The varied presentations could also be accounted for by the different medical history between the two individuals, timeline of developing symptoms, or response to treatment.

## Conclusions

This case is unique because it is the first reported case of familial VGKC autoimmune encephalitis. Both brothers presented with a nonspecific prodrome of headache and fatigue with neuropsychiatric symptoms. Patient 1 had more motor symptoms and responded well to initial treatment. Patient 2 had a more indolent course developing symptoms over weeks, with more psychiatric symptoms; he required rituximab for further immunosuppression. The differences between the presentations of these brothers could be explained by a multifactorial autoimmune pathophysiology: similar yet different genetics and antigen processing and presentation; perhaps, different antigen targets on the VGKC channel, distinct medical history, same family, but different family stressors.

Prompt etiologic diagnosis of encephalitis may lead to improved clinical outcomes. This case is important because autoimmunity is an emerging cause of encephalitis in children. The incidence and prevalence of autoimmune encephalitis are similar to infectious encephalitis. If unrecognized or untreated, brain damage can occur. Once meningitis and other infectious causes are ruled out, an autoimmune cause must be considered. Response to treatment should be noted, as relapses are variable and can be managed with different therapies. Following established protocols, clear communication with families, using multidisciplinary rehabilitation, and follow-up when necessary can help reduce disease burden and improve long-term outcomes.
